# The evolving world of pseudoenzymes: proteins, prejudice and zombies

**DOI:** 10.1186/s12915-016-0322-x

**Published:** 2016-11-11

**Authors:** Patrick A. Eyers, James M. Murphy

**Affiliations:** 1 Department of Biochemistry, Institute of Integrative Biology, University of Liverpool, Liverpool, L69 7ZB UK; 2 The Walter and Eliza Hall Institute of Medical Research, 1G Royal Parade, Parkville, VIC Australia; 3 Department of Medical Biology, University of Melbourne, Parkville, VIC Australia

## Abstract

Pseudoenzymes are catalytically deficient variants of enzymes that are represented in all major enzyme families. Their regulatory functions in signalling pathways are shedding new light on the non-catalytic functions of active enzymes, and are suggesting new ways to target cellular signalling mechanisms with drugs.

## What is a pseudoenzyme?

A pseudoenzyme contains a protein domain with a fold that resembles, or is predicted to resemble, a catalytically active, conventional protein domain counterpart. However, the pseudoenzyme possesses vestigial or zero catalytic activity owing to the absence of key catalytic amino acids or motifs. Such proteins have been long acknowledged to exist within proteomes and have also been termed nonenyzmes [1]. In a sense, pseudoenzymes can be considered to be ‘zombie’ versions of proteins: ‘undead’ in the sense that they still perform important cellular functions, but at the same time ‘dead’ since they possess negligible enzymatic activity relative to their catalytically active cousins.

## How are pseudoenzymes identified?

Initially, by sequence comparisons. As genomes began to be sequenced, and the analytical tools of computational biology matured, accurate alignment and comparison of deduced amino acid sequences revealed common ‘signatures’ that could be compared and contrasted between all polypeptides. The major cell signaling enzyme families led the way in this regard, with early efforts using manual alignment generating clear patterns of amino acid conservation in classic enzyme families (for example amongst the protein kinases [2]). These patterns often explain how an enzyme works or is likely to be regulated [3, 4]. They may include, for example, the presence of a metal-binding motif or a conserved network of amino acids that form a catalytic signature.

What has become apparent now that thousands and thousands of genomes have been sequenced is that they contain, within every enzyme family where we look, divergent examples of proteins that are clearly part of large enzyme families but for which basic enzyme function (increasing the rate of a chemical reaction) is predicted to be lacking. These ‘dead’ enzymes have amino acid sequence deviations from the catalytic residues of counterparts that would be predicted to confer enzyme function—or, in an experimentally ideal world, where such function has been quantified biochemically. Thus, classification of pseudoenzymes is easiest for enzyme domains for which we have an accumulated knowledge of catalytic mechanism.

Undoubtedly, however, many pseudoenzymes have yet to be classed as such, and there are probably many ‘enzymes’ that are actually pseudoenzymes; we just haven’t confirmed this yet experimentally. Notably, because there are distinct sequence deviations that might compromise a cousin enzyme’s catalytic activity to generate a pseudoenzyme, predicting whether a protein is catalytically deficient simply from inspection of its sequence, not to mention the complexities of subsequent experimental validation, remains an enormous challenge.

## So what does it take to be confident you have identified a pseudoenzyme?

The best studies of pseudoenzymes attempt to make structure:function predictions and then test them using structural biology, biochemical, cellular and organism-based analysis. Areas of enzymology that have led this endeavor include studies of the kinases (which in terms of simple classification by mechanism are ATP-binding phosphotransferase enzymes with specificity for either small molecules or proteins [5]) and the vast numbers of metabolic enzymes analysed over the course of biochemical history. In an ideal study, comparative biochemical analysis of each (pseudo)enzyme should occur, although this is a very time-consuming process and rarely possible in practice (what if no turnover is detected; have the ‘correct’ conditions, cofactors and substrates been tested; what if there are no apparent catalytically active counterparts for comparison?).

## How do catalytically inert enzymes impact on signalling networks?

**Fig. 1.**
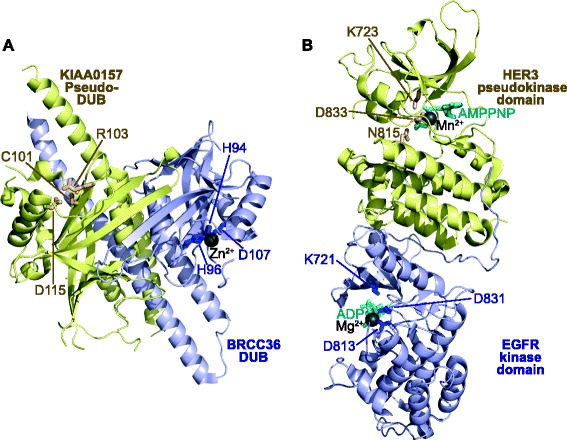
Pseudoenzymes can allosterically regulate a partner protein’s catalytic activity. **a** The pseudo-deubiquitinase (*DUB*), KIAA0157 (*yellow*), which lacks a canonical Zn-binding motif (residues shown as *sticks* and labelled), binds to the canonical DUB, BRCC36 (*blue*), to promote its DUB activity. BRCC36 binds Zn^2+^ (*grey sphere*) via the labelled residues (shown as *blue sticks*). The KIAA0157–BRCC36 heterodimeric complex assembles into a higher order “superdimer”, which is an active DUB. PDB accession 5CW3 [11]. **b** The pseudokinase domain of HER3 (*yellow*) binds ‘head-to-tail’ and allosterically regulates the activation of the conventional protein tyrosine kinase EGFR (*blue*). While each domain can accommodate and bind to a nucleotide (*cyan*) and a divalent cation (*grey spheres*), HER3 exhibits defective catalytic activity owing to substitution of the catalytic Asp acid residue to an Asn at position 815 (*yellow sticks*). In contrast, EGFR contains conventional catalytic residues (*blue sticks*, labelled), which allow it to phosphorylate substrates on tyrosine residues. PDB accession 4RIW [31]. Cartoons were drawn using Pymol

Because of the prejudice that focused attention on the catalytic functions of enzymes in signalling pathways, for a long time pseudoenzymes were considered to be dead—and therefore evolutionary remnants or bystanders in cell signalling networks. Contrary to this view, however, pseudoenzymes have now emerged as crucial players operating with an impressive diversity of mechanisms that we are only beginning to understand. Explaining how pseudoenzymes fit into our current paradigms of cell signalling is an important challenge and crucial to developing ‘systems’ models of signal transduction. We are now realizing that enzymes that regulate signalling, such as kinases, phosphatases, proteases, GTPases, ubiquitin modifying enzymes, acetylases and sulfotransferases, can have many functional properties other than catalysis, and these can be shared by conventional enzymes and their pseudoenzyme counterparts. For example, they can function as dynamic scaffolds that nucleate signalling, or as competitors in canonical signalling pathways, or as direct modulators (activators or inhibitors) of enzymes [6]. Two examples of activation by pseudoenzymes can be found in Fig. 1. Direct modulation of this sort appears to be particularly important for pseudokinases [7], which are amongst the best-studied pseudoenzymes and the subject of research in the authors’ laboratories.

## How did pseudoenzymes evolve?

We await a formal analysis of pseudoenzyme evolution but it is likely that pseudoenzymes evolved from enzyme counterparts through gene duplication that created an opportunity for additional copies to diverge in sequence from ancestral enzymes in the absence of selective pressure at the catalytic motifs normally required for canonical enzyme function. Thus, a conserved fold or scaffold can evolve new ‘pseudoenzyme’ functions by ‘repurposing’ a classic enzyme fold because the selective pressure to maintain active site geometry (to support a catalytic cycle of substrate binding, transition state, catalysis and substrate release) is relieved. We should not rule out, however, at least in some cases, the formal possibility that enzymes may actually have evolved from pseudoenzymes, and there are some interesting examples of likely pseudoenzyme:enzyme switches amongst the protein kinases [6].

## Have all families of enzyme evolved pseudoenzyme paralogs?

This is a good question, and very much in need of a comprehensive bioinformatic analysis. However, in essentially any family of enzymes for which we have a basic understanding of the catalytic residues, we rarely fail to find counterparts with deviations in catalytic residues for which we would subsequently predict a catalytic deficit. On the other hand, it should be stressed that we rarely have biochemical evidence that pseudoenzymes do indeed lack catalytic activity, either because we do not know what their physiological substrate would likely be or because in-depth experimental examination is pending. However, for several of the best-studied enzyme families involved in signalling (protein kinases, phosphatases, deubiquitinases, proteases), well studied examples of pseudoenzymes occur throughout nature, with biochemical, structural and cellular experimental evidence existing to back this up [6, 8–11].

## How do structural features of the original enzymes suit new pseudoenzyme functions?

**Fig. 2.**
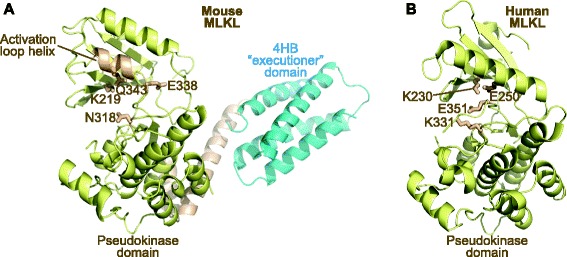
Pseudoenzymes can adopt multiple structural conformations, permitting them to function as molecular switches. **a** The pseudokinase domain of mouse MLKL was crystallised in an open (equivalent to catalytically inactive) conformation in which the activation loop adopted an unusual helix that buttresses against, and shuns, the αC helix. Counterparts of the catalytic residues in conventional protein kinases (K219, N318, E338), and the K219-interacting Q343 from the activation loop helix, are shown as *yellow sticks*. PDB accession 4BTF [18]. **b** The structure of the human MLKL pseudokinase domain crystallised in a distinct closed conformation which resembles that of an active conventional protein kinase, suggesting that MLKL has evolved to function as a catalytically inactive conformational switch. Counterparts of the catalytic residues in conventional protein kinases (K230, K331, E351) are shown as *yellow sticks*. The canonical αC helix glutamic acid (E250), rather than the activation loop residue observed in the mouse structure, interacts with K230, as is typical of active protein kinase structures. PDB accession 4MWI [14]. Accompanying mutational analyses illustrated that nucleotide binding by MLKL is mediated by different pseudoactive site determinants and that K219 (mouse) and K230 (human) have evolved unexpected functions to permit nucleotide binding, which might drive or inhibit a switch mechanism that controls release of the MLKL four-helix bundle (*4HB*) domain (shown in **a**) to induce cell death by necroptosis [14, 18]. Cartoons drawn using Pymol

We have already mentioned that catalytically active enzymes can also serve important functions as modulators of signalling through non-catalytic functions [12, 13]. This leads us to believe that enzyme structures are predisposed to mediating interactions with protein or metabolite ligands and thus these folds are the ideal templates for nature to repurpose for entirely new functions (Figs. 1 and 2). While pseudoenzymes typically arise from subtle deviations in amino acid sequence relative to catalytically active counterparts, the extent and manner of the impact of these deviations on the structure differs for each pseudoenzyme. Again, we know most about the protein pseudokinases, where widespread loss or occasional unconventional modes of nucleotide binding have evolved in the absence of selective pressure to maintain active site geometry for catalysis. An example is the pseudokinase MLKL, illustrated in Fig. 2 [10, 14]. In turn, this creates a new scenario in which the stable fold of a pseudoenyzme can be exploited for a new biological function, such as mediating protein interactions via an allosteric site or repurposing of the substrate binding site to regulate the signalling output of a partner protein by modulating its cellular localization, activation or catalytic activity (e.g. [15, 16]).

## How do pseudoenzymes adapt for their new roles?

Structural and biochemical studies over the past decade have allowed us to grasp a greater appreciation of the many mechanisms by which pseudoenzymes can adapt the conventional enzyme fold to mediate new functions. For example, in the case of pseudophosphatases, the adaption is thought to create a tight-binding ‘phosphate trap’ that prevents substrates (phosphorylated proteins) from being dephosphorylated and/or shields this phosphorylated epitope from the cellular machinery [17]. This might then prolong a signalling event or change the subcellular distribution of the target protein, including networks of proteins normally regulated by the pseudosubstrate [6]. For pseudokinases, there appears to be a rich vein of adaptive capacity. As described above, conserved deviations of key residues within the active site appear to relieve selective pressures on active site geometry [14, 18], allowing for evolution of conformational changes and additional allosteric functions.

## How do studies of pseudoenzymes shed light on the mechanisms of active enzymes?

Recent studies support the idea that insights from studies of pseudoenzymes can provide a window into understanding the non-catalytic functions of conventional enzymes [12, 13, 19]. On the whole, it is conformation (rather than catalytic output) that is most critical for the regulated activation and inactivation cycles that most enzymes undergo as part of homeostatic mechanisms. This observation is echoed in studies of multiple pseudokinases, which can be trapped in conformations synonymous with either active or inactive versions of their catalytically active kinase cousins (see, for example, [14, 18]; Fig. 2). Such conformational changes can be promoted by, for example, allosteric interactions with binding partners or post-translational modifications such as phosphorylation. In most cases we do not understand how these different conformations lend themselves to distinct functions, but their existence is generally supportive of the emerging idea that pseudoenzyme functions must also be controlled, suggesting that the different conformations represent the ‘on/off’ phases of a molecular switch (Fig. 2).

In addition, knowledge of catalytic mechanisms can be obtained from studies of conventional enzymes and confirmation of mechanism can come from (pseudo)enzyme comparisons. This is particularly apparent in the pseudophosphatases, in which a solitary amino acid substitution can restore the highly conserved Cys-based phosphatase catalytic mechanism that is lost in the pseudophosphatase [20]). By contrast it is comparatively difficult to turn pseudokinases back into enzymatically active kinases, suggesting that the evolutionary events exploited for their new roles are too numerous to allow facile reversion to the enzyme-mediated mode of signalling of their ancestor. Interestingly, we are learning from comparisons of signalling pseudoenzymes and enzymes that catalytic output from enzymes varies over many orders of magnitude and that pseudoenzymes may also fine-tune the activity of enzyme interaction partners. This might dampen down catalytic output when it is a potential liability, for example where suppression of the catalytic activity of the canonical tyrosine kinase domain of the Janus kinase (JAK) by the neighbouring pseudokinase domain depresses proliferative signalling that could otherwise be tumourigenic [21, 22]. Most enzymes are subject to many layers of regulation, but as we have learned from oncogenic protein kinases (for example BRAF), a single amino acid switch that locks them into a ‘super enzyme’ mode can be lethal; thus, it may be an evolutionary advantage that some types of pseudoenzymes are not readily reactivated by reversion mutations.

## What are the known functions of pseudoenzymes?

Their functions encompass major regulatory roles in all areas of biology and across life from prokaryotes to eukaryotes. Although several reviews discuss pseudoenzymes in cell signalling [6–9], and we know most about the pseudoenzymes that have evolved in the kinase and phosphatase families, this area is ripe for further exploration. For example, in the tyrosine phosphatase family, multiple human pseudophosphatase domains have evolved [23], with a catalytically inert pseudophosphatase present in parallel within the same polypeptide alongside a canonical phosphatase domain containing assayable catalytic activity. This situation is also found in some pseudokinase domain-containing proteins (for example Janus kinases; GCN2), in which the pseudokinase domain is the key regulatory element that controls the catalytic output through an adjacent canonical kinase domain. Why has this arrangement of pseudoenzyme and enzyme domains proved so popular? The answer, somewhat surprisingly, remains unknown.

## Which are the pseudoenzymes of most interest at present?

It is hard to say currently where our research directions should specifically be focused, since the field is so young, and in reality, scientists are in desperate need of new tools and technology to identify, classify, evaluate and uncover the biology of the pseudoenzymes. However, it might be particularly pertinent to study pseudoenzymes in more primitive organisms since these may reveal ancestral roles that have been co-opted throughout evolution (see, for example, [1]). In addition, quite recent findings demonstrating that both intracellular and extracellular phosphorylation-dependent signalling can be driven by pseudokinases and kinase co-operation are very exciting, consistent with a major function of pseudoenzymes in fine-tuning the output of catalytically active enzymes [16, 24]. In our laboratories, we do of course have our particular research favourites, and these generally represent the analysis of specific human pseudokinases. For example, we think that MLKL, which controls necroptosis through a switch-like mechanism [14, 18] and the Tribbles pseudokinases, which modulate protein ubiquitination [15, 25], are of outstanding interest as models for understanding basic signalling mechanisms by pseudoenzymes. Both of these exemplify the vast and varied manner in which pseudoenzymes can modulate cell signalling outputs via roles in mediating protein interactions, including nucleating and controlling the output from multiprotein signalling complexes.

## Can pseudoenzymes be therapeutically targeted?

This is a really important question and the answer is almost certainly yes. Indeed, based on the direction of pharma and some of the patent literature, it is certain that scientists are attempting to target pseudoenzymes, both knowingly and (thought provokingly) unknowingly. The likely conservation of structural fold, and a reliance on protein–protein interactions for outputs, mean that pseudoenzymes might be targeted therapeutically in essentially the same ways as other enzymes (see, for example, [26–28]). Indeed, in the case of pseudokinases, it was found that promiscuous inhibitors that target the ATP-binding cleft of conventional protein kinases could also bind the equivalent site in pseudokinases despite most of the examined pseudokinases exhibiting no detectable ATP-binding capacity [10]. The difference is that pseudoenzymes are likely to have degraded active sites, so that although many drug families might be re-purposed to target pseudoenzymes, new programs of drug discovery are urgently needed to specifically modulate them. The deviations in ‘pseudoactive’ site geometry raise the attractive idea that pseudoenzymes might have evolved distinct features that will enable their specific targeting by small molecules in preference to their catalytically active cousins. Of particular interest in the field of signalling is that of the >50 protein pseudokinases found in vertebrates [7], half have already been implicated in one disease or another [6]. This furnishes enough potential drug candidates to keep pharma busy for years, and we predict that the pseudophosphatase, pseudoprotease and pseudo-deubiquitinase fields are also likely to rise to pharmaceutical prominence over the next decade. One prominent example of a promising pseudokinase drug target is the Janus kinase (JAK) family of pseudokinase domains, which are master regulators of the catalytic output of JAK-driven signalling pathways central to blood cell function. Indeed, the V617F mutation in the JAK2 pseudokinase domain is known to promote the catalytic activity of the adjacent tyrosine kinase domain [21, 29]. As a result, this pseudokinase domain mutation is causative of the myeloproliferative neoplasm polycythemia vera, making this a very exciting potential drug target [22].

## What are the most important issues in the future of pseudoenzyme research?

While we have made a lot of progress over the past 10 years, we still lack knowledge of the cellular functions performed by most pseudoenzymes and their evolutionary origins, including patterns of conservation across various genomes. For some pseudoenzyme/enzyme families this understanding is well on the way, but is potentially confounded by nature’s repurposing of distinct structural folds, which are not always obvious from primary structures of proteins, especially for convergent functions [30]. Even within closely related pseudoenzymes families (such as the three Tribbles pseudokinases, Trib1, Trib2 and Trib3 and their obscure pseudokinase cousin, SgK495) different biological and biochemical functions have been attributed to each pseudoenzyme in mammals. This highlights a major challenge facing those in the field, which is the necessity to study each and every pseudoenzyme in detail, using structural biology, biochemistry, cell biology and in vivo models, to deduce mechanism of action and biological function. As more and more pseudoenzymes are assigned functions in signalling pathways and become foci for drug discovery, we predict that the suite of available tools and methodologies to rigorously interrogate pseudoenzymes will coordinately grow, ensuring that the community continues to expand and thrive.

## References

[1] Todd AE, Orengo CA, Thornton JM (2002). Sequence and structural differences between enzyme and nonenzyme homologs. Structure.

[2] Hanks SK, Quinn AM, Hunter T (1988). The protein kinase family: conserved features and deduced phylogeny of the catalytic domains. Science.

[3] Manning G, Whyte DB, Martinez R, Hunter T, Sudarsanam S (2002). The protein kinase complement of the human genome. Science.

[4] Manning G, Plowman GD, Hunter T, Sudarsanam S (2002). Evolution of protein kinase signaling from yeast to man. Trends Biochem Sci.

[5] Kannan N, Taylor SS, Zhai Y, Venter JC, Manning G (2007). Structural and functional diversity of the microbial kinome. PLoS Biol.

[6] Reiterer V, Eyers PA, Farhan H (2014). Day of the dead: pseudokinases and pseudophosphatases in physiology and disease. Trends Cell Biol.

[7] Eyers PA, Murphy JM (2013). Dawn of the dead: protein pseudokinases signal new adventures in cell biology. Biochem Soc Trans.

[8] Adrain C, Freeman M (2012). New lives for old: evolution of pseudoenzyme function illustrated by iRhoms. Nat Rev Mol Cell Biol.

[9] Boudeau J, Miranda-Saavedra D, Barton GJ, Alessi DR (2006). Emerging roles of pseudokinases. Trends Cell Biol.

[10] Murphy JM, Zhang Q, Young SN, Reese ML, Bailey FP, Eyers PA, Ungureanu D, Hammaren H, Silvennoinen O, Varghese LN (2014). A robust methodology to subclassify pseudokinases based on their nucleotide-binding properties. Biochem J.

[11] Zeqiraj E, Tian L, Piggott CA, Pillon MC, Duffy NM, Ceccarelli DF, Keszei AF, Lorenzen K, Kurinov I, Orlicky S (2015). Higher-order assembly of BRCC36-KIAA0157 is required for DUB activity and biological function. Mol Cell.

[12] Kung JE, Jura N (2016). Structural basis for the non-catalytic functions of protein kinases. Structure.

[13] Shaw AS, Kornev AP, Hu J, Ahuja LG, Taylor SS (2014). Kinases and pseudokinases: lessons from RAF. Mol Cell Biol.

[14] Murphy JM, Lucet IS, Hildebrand JM, Tanzer MC, Young SN, Sharma P, Lessene G, Alexander WS, Babon JJ, Silke J (2014). Insights into the evolution of divergent nucleotide-binding mechanisms among pseudokinases revealed by crystal structures of human and mouse MLKL. Biochem J.

[15] Murphy JM, Nakatani Y, Jamieson SA, Dai W, Lucet IS, Mace PD (2015). Molecular mechanism of CCAAT-enhancer binding protein recruitment by the TRIB1 pseudokinase. Structure.

[16] Zeqiraj E, Filippi BM, Goldie S, Navratilova I, Boudeau J, Deak M, Alessi DR, van Aalten DM (2009). ATP and MO25alpha regulate the conformational state of the STRADalpha pseudokinase and activation of the LKB1 tumour suppressor. PLoS Biol.

[17] Tonks NK (2009). Pseudophosphatases: grab and hold on. Cell.

[18] Murphy JM, Czabotar PE, Hildebrand JM, Lucet IS, Zhang JG, Alvarez-Diaz S, Lewis R, Lalaoui N, Metcalf D, Webb AI (2013). The pseudokinase MLKL mediates necroptosis via a molecular switch mechanism. Immunity.

[19] Reynolds SL, Fischer K (2015). Pseudoproteases: mechanisms and function. Biochem J.

[20] Wishart MJ, Dixon JE (1998). Gathering STYX: phosphatase-like form predicts functions for unique protein-interaction domains. Trends Biochem Sci.

[21] James C, Ugo V, Le Couedic JP, Staerk J, Delhommeau F, Lacout C, Garcon L, Raslova H, Berger R, Bennaceur-Griscelli A (2005). A unique clonal JAK2 mutation leading to constitutive signalling causes polycythaemia vera. Nature.

[22] Silvennoinen O, Hubbard SR (2015). Molecular insights into regulation of JAK2 in myeloproliferative neoplasms. Blood.

[23] Tonks NK (2006). Protein tyrosine phosphatases: from genes, to function, to disease. Nat Rev Mol Cell Biol.

[24] Cui J, Xiao J, Tagliabracci VS, Wen J, Rahdar M, Dixon JE (2015). A secretory kinase complex regulates extracellular protein phosphorylation. Elife.

[25] Bailey FP, Byrne DP, Oruganty K, Eyers CE, Novotny CJ, Shokat KM, Kannan N, Eyers PA (2015). The Tribbles 2 (TRB2) pseudokinase binds to ATP and autophosphorylates in a metal-independent manner. Biochem J.

[26] Dhawan NS, Scopton AP, Dar AC (2016). Small molecule stabilization of the KSR inactive state antagonizes oncogenic Ras signalling. Nature.

[27] Foulkes DM, Byrne DP, Bailey FP, Eyers PA (2015). Tribbles pseudokinases: novel targets for chemical biology and drug discovery?. Biochem Soc Trans.

[28] Hildebrand JM, Tanzer MC, Lucet IS, Young SN, Spall SK, Sharma P, Pierotti C, Garnier JM, Dobson RC, Webb AI (2014). Activation of the pseudokinase MLKL unleashes the four-helix bundle domain to induce membrane localization and necroptotic cell death. Proc Natl Acad Sci U S A.

[29] Varghese LN, Ungureanu D, Liau NP, Young SN, Laktyushin A, Hammaren H, Lucet IS, Nicola NA, Silvennoinen O, Babon JJ (2014). Mechanistic insights into activation and SOCS3-mediated inhibition of myeloproliferative neoplasm-associated JAK2 mutants from biochemical and structural analyses. Biochem J.

[30] Todd AE, Orengo CA, Thornton JM (2002). Plasticity of enzyme active sites. Trends Biochem Sci.

[31] Littlefield P, Liu L, Mysore V, Shan Y, Shaw DE, Jura N (2014). Structural analysis of the EGFR/HER3 heterodimer reveals the molecular basis for activating HER3 mutations. Sci Signal.

